# Higher waist circumference is associated with increased likelihood of female infertility: NHANES 2017-2020 results

**DOI:** 10.3389/fendo.2023.1216413

**Published:** 2023-10-20

**Authors:** Ying-Hua Yin, Su-Yu Zhou, Dong-Fang Lu, Xiu-Ping Chen, Bo Liu, Shan Lu, Xiao-Dong Han, Ai-Hua Wu

**Affiliations:** ^1^The Second Clinical Medical College, Guangzhou University of Chinese Medicine, Guangzhou, China; ^2^State Key Laboratory of Dampness Syndrome of Chinese Medicine, Guangzhou, China; ^3^Center for Reproductive Medicine, Guangdong Hospital of Traditional Chinese Medicine, Guangzhou, China

**Keywords:** waist circumference, female infertility, NHANES, cross-sectional study, reproductive ability

## Abstract

**Background:**

Waist circumference can be used as an anthropometric measure to assess central obesity and is easier and more convenient than the waist-to-hip ratio in identifying the risk of obesity and medical problems. Most studies showing an association between obesity and infertility in women have used BMI to measure obesity. Our goal was to examine any potential association between waist circumference and infertility.

**Methods:**

This cross-sectional study, which formed part of the National Health and Nutrition Examination Survey (NHANES), comprised women ages 18 to 45 between 2017 and 2020. Participants without waist circumference data or information on infertility were removed from the study. The independent relationship between waist circumference and infertility was investigated using weighted binary logistic regression and subgroup analysis.

**Results:**

We investigated 1509 participants and discovered that the prevalence of infertility rose as the WC trisection rose. (tertile 1, 7.55%; tertile 2, 10.56%; tertile 3, 15.28%; trend < 0.001). Multivariate logistic regression showed that after total adjustment, higher WC levels were associated with an increased likelihood of infertility in women (OR1.02; 95% CI 1.01-1.03), and There was a 2% rise in the incidence of infertility for every unit (cm) increased WC. Subgroup analysis and interaction tests showed no significant dependence of the effects of marital status, diabetes, hypertension, and high cholesterol on the association between WC and infertility (*p* for all interaction tests > 0.05). The inflection point of the positive non-linear relationship between WC and infertility was 116.6 cm.

**Conclusion:**

Excessive waist circumference assessment may increase the probability of infertility, and more attention should be paid to the management of waist circumference should be given more attention.

## Introduction

Infertility is a condition that has historically been described as the inability to conceive successfully after more than 12 months of regular, unprotected sexual activity or due to a decrease in one’s ability to reproduce either alone or with a partner (1). The World Health Organization recognizes infertility as a significant public health issue that affects up to 186 million individuals globally. It has a prevalence of up to 15% among couples of reproductive age (2–5). According to estimates, more than half of infertile women experience stress due to significant psychological issues such as melancholy, anxiety, and social dysfunction (6). Their overall health is affected to some extent (7).

Overweight and obesity have evolved into a global public health issue as the prevalence of obesity rises year over year (8). Global obesity prevalence among women is projected to exceed 21% by 2025 (9). Overweight represents the process of excessive accumulation of fat and is classified as BMI > 25 kg/m^2^ (“overweight”) and BMI > 30 kg/m^2^ (“obese”) (10). Numerous studies have shown that it increases the risk of many common diseases, including endometrial and postmenopausal breast cancer, diabetes mellitus, hypertension, cardiovascular disease, gallstones, non-alcoholic fatty liver disease, dyslipidemia, and infertility due to ovulatory disorders (11, 12). In addition, obesity has a negative impact on human reproduction, including menstrual abnormalities, infertility, reduced live birth rates, and pregnancy complications (13, 14).

Although measuring the prevalence of obesity by BMI has become a mainstream test, it lacks an adequate benchmark for sensitivity and specificity (15, 16). Although the majority of obesity as measured by BMI may have stabilized in some countries, the prevalence of abdominal obesity as measured by waist circumference has generally increased, and the dynamics of obesity phenotypes over time suggest that most evidence indicates that BMI has limitations in identifying obesity phenotypes that convey the most significant health risks (17). As the prevalence of abdominal obesity increases, BMI is no longer sufficient to predict current obesity prevalence trends and waist circumference is better than BMI to detect the majority of abdominal obesity (18), In addition, there may be differences in the prevalence of infertility among obese individuals diagnosed based on waist circumference or body mass index if body mass index (BMI) rather than waist circumference is used for the analysis. so in this study, we investigate the inclusion of waist circumference in obesity surveillance studies.

In addition, many professionals advocate the inclusion of waist circumference in the analysis of obesity surveillance because of the increasing prevalence of abdominal obesity worldwide (19). Waist circumference (WC) provides a simple measure of obesity index and fat distribution (20); according to a study, many people in poor health also have high waist circumference values (21–23). Research has revealed that BMI is linked to several reproductive diseases, including endometriosis, pre-eclampsia, and miscarriage (24), While there are fewer studies on waist circumference and reproductive disorders.

Therefore, in this cross-sectional study of women in couples having infertility evaluation and treatment, we looked into the relationship between WC and infertility prevalence to further our understanding of this field.

## Materials and methods

### Study population

**Figure 1:**
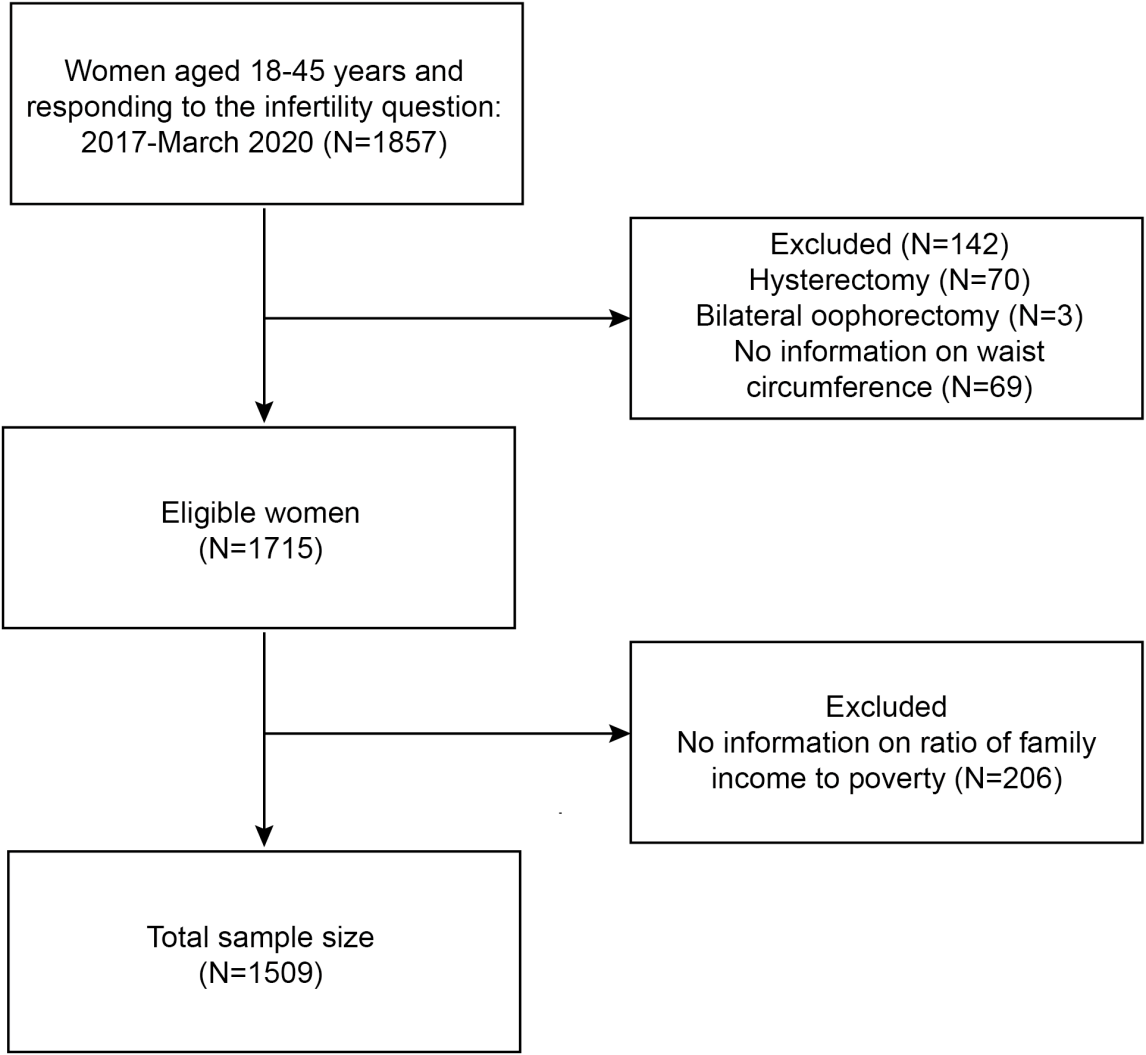
Flowchart of the sample selection from NHANES 2017-March 2020.

NHANES is a study that the National Center for Health Statistics (NCHS) oversees that attempts to assess the health and nutritional status of the population in the United States. The public may access all NHANES data at https://www.cdc.gov/nchs/nhanes/. The sample used for the NHANES is fairly representative because of the stratified multistage probability sampling technique used in the research design. NHANES data are made available on a two-year cycle to the general public. Women who participated in the NHANES during the March 2017–2020 cycle provided the data for this cross-sectional research. Women aged 18-45 answered: ‘Have you ever tried to get pregnant for at least one year without getting pregnant?’ (n = 1857) were included in the study. Women who underwent hysterectomy (n = 70), underwent bilateral oophorectomy (n = 3), and lacked information on waist circumference (n = 69) and household income to poverty ratio (n = 206) were excluded. Finally, 1509 women qualified for analysis (**Figure 1**). Each participant in the NHANES study offered written permission, which also was evaluated and approved by the NCHS Research Ethics Review Committee.

### Exposure and ending definition

In this study, waist circumference was considered an exposure variable, and WC data were obtained from the NHANES 2017-March 2020 Pre-Pandemic Examination Data (P BMX). Detailed measurements of WC are shown on the NHANES website.

Self-reported infertility data were obtained from the NHANES Reproductive Health Questionnaire (RHQ). The presence of infertility was assessed based on the following question: “Have you ever tried to get pregnant for at least one year without getting pregnant? Women who answered “yes” were considered to be infertile, and those who answered “no” were considered to be fertile.

### Covariates

Covariates that may affect the connection between waist circumference and infertility were also included in our investigation. Continuous variables included Age (years), Ratio of family income to poverty, Minutes of sedentary activity, and Meals from fast food or pizza place. Total Cholesterol, Direct HDL-Cholesterol. Categorical variables included Race (Mexican American/Other Hispanic/Non-Hispanic White/Non-Hispanic Black/Other Races), Marital status (Married with Partner/Divorced/Never married), Educational level (Less than 9th grade/9th-11th grade/High school or GED/Some college or AA degree/College graduate or above), Diabetes. Hypertension, and high cholesterol level, all of these variables were extracted from NHANES demographics, screening data, questionnaires, and laboratory measurements. All of their detailed measurement procedures are publicly available at https://www.cdc.gov/nchs/nhanes/.

### Statistical analysis

As instructed by the Centers for Disease Control and Prevention (CDC), we conducted all statistical analyses using R (http://www.r-project.org) and EmpowerStats (http://www.empowerstats.com), with the statistical significance threshold set at *p* < 0.05. The NCHS analysis criteria produced all estimates, and the sample weights were created to satisfy the NHANES objectives. The specific weighting strategy was to use waist circumference as a continuous variable to delineate the tertiles. The composition of percentages represents categorical variables, while the mean of standard deviations is used to describe continuous variables. Differences between groups divided by WC triads were assessed using weighted Student’s t-tests (for continuous variables) or weighted chi-square tests (for categorical variables). The independent connection between WC and infertility was investigated in three distinct models using multifactorial logistic regression. Covariate adjustments were not applied in model 1 at all. Race and age adjustments were made to Model 2. Age, race, educational level, Ratio of family income to poverty, marital status, Minutes of sedentary activity, Meals from fast food or pizza place, Total Cholesterol, Direct HDL-Cholesterol, hypertension, and diabetes adjustments were made to Model 3. In addition to subgroup studies stratified by marital status, diabetes, hypertension, and high cholesterol levels, smoothed curve fitting and threshold effects analysis were performed to investigate the nonlinear association between WC and infertility.

### Baseline characteristics of participants

A total of 1,509 female participants aged 18-45 years were included in this study, and their weighted demographic baseline characteristics are shown in **Table 1**. The waist circumference range in the tertile 1-3 was 56.4-84.3, 84.4-102.8, and 102.9-178, respectively. Waist circumference in different tertile ranges for age, race, marital status, education, diabetes mellitus, hypertension, high cholesterol levels, infertility, Ratio of family income to poverty, Minutes of sedentary activity, Meals from fast food or pizza place, Total Cholesterol, and Direct HDL-Cholesterol differed with statistically significant (all *p* < 0.05). The prevalence of infertility in tertile 1, tertile 2, and tertile 3 was 7.55%, 10.56%, and 15.28%, respectively, and the majority of infertility increased with increasing WC tertile.

**Table 1:** Baseline characteristics of the study population according to Waist Circumference tertiles.

Waist Circumference (cm)	Tertile1 (56.4-84.3)	Tertile2 (84.4-102.8)	Tertile3 (102.9-178)	*p* value
Age(years)	28.81 ± 7.92	31.63 ± 8.10	32.78 ± 8.02	<0.001
Race (%)				<0.001
Mexican American	46 (9.15%)	98 (19.52%)	73 (14.48%)	
Other Hispanic	54 (10.74%)	52 (10.36%)	37 (7.34%)	
Non-Hispanic White	162(32.21%)	139(27.69%)	167 (33.13%)	
Non-Hispanic Black	109(21.67%)	129(25.70%)	172 (34.13%)	
Other Races	132(26.24%)	84 (16.73%)	55 (10.91%)	
Marital status(%)				<0.001
Married with Partner	226(52.68%)	281(61.09%)	260 (54.97%)	
Divorced	26 (6.06%)	47 (10.22%)	48 (10.15%)	
Never married	177(41.26%)	132(28.70%)	165 (34.88%)	
Education level(%)				<0.001
Less than 9th grade	12 (2.80%)	20 (4.35%)	15 (3.17%)	
9-11th grade	35 (8.16%)	42 (9.13%)	53 (11.21%)	
High school or GED	75 (17.48%)	83 (18.04%)	111 (23.47%)	
Some college or AA degree	149(34.73%)	185(40.22%)	201 (42.49%)	
College graduate or above	158(36.83%)	130(28.26%)	93 (19.66%)	
Diabetes(%)				<0.001
Yes	11 (2.19%)	17 (3.39%)	49 (9.72%)	
No	492(97.81%)	485(96.61%)	455 (90.28%)	
Hypertension(%)				<0.001
Yes	25 (4.97%)	41 (8.17%)	106 (21.03%)	
No	478(95.03%)	461(91.83%)	398 (78.97%)	
high cholesterol level(%)				<0.001
Yes	36 (7.16%)	66 (13.15%)	85 (16.87%)	
No	467(92.84%)	436(86.85%)	419 (83.13%)	
Infertility(%)				<0.001
Yes	38 (7.55%)	53 (10.56%)	77 (15.28%)	
No	465 (92.45%)	449 (89.44%)	427 (84.72%)	
Ratio of family income to poverty	2.52 ± 1.74	2.36 ± 1.65	2.05 ± 1.45	<0.001
Minutes sedentary activity	321.40 ± 185.00	365.08 ± 640.69	417.45 ± 775.79	0.031
Meals from fast food or pizza place	2.12 ± 2.48	2.33 ± 3.03	24.72 ± 471.25	0.001
Total Cholesterol (mmol/L)	4.31 ± 0.80	4.66 ± 0.98	4.68 ± 0.89	<0.001
Direct HDL-Cholesterol (mmol/L)	1.63 ± 0.38	1.44 ± 0.40	1.28 ± 0.33	<0.001

Mean+SD for continuous variables: p value was calculated by the weighted linear regression model.

% for Categorical variables:p value was calculated by the weighted chi-square test.

### A greater WC is linked to a higher risk of infertility

A positive correlation between WC and infertility prevalence can be observed in **Table 2**. This positive association remained stable in the fully adjusted model (model 3) (OR1.02; 95% CI 1.01-1.03). It showed that each unit increase in WC was associated with a 2% increase in infertility risk. We also converted WC from a continuous variable to a categorical (triplet) for sensitivity analysis. A significant increase of 121% in the likelihood of developing infertility was observed in tertile three compared to the lowest WC tertile (tertile 1). However, the difference between tertile 1 and tertile 2 was not statistically significant (OR1.40; 95% CI 0.79-2.49).

**Table 2:** A greater WC is linked to a higher risk of infertility.

	OR(95%CI), *p* value		
	Model 1^1^ (n = 503)	Model 2^2^ (n = 502)	Model 3^3^ (n = 504)
Infertility			
Waist Circumference	1.02 (1.01, 1.03) <0.01	1.02 (1.01, 1.03) <0.01	1.02 (1.01, 1.03) <0.01
Categories			
Tertile1	Reference	Reference	Reference
Tertile2	1.44 (0.93, 2.23) 0.09	1.32 (0.84, 2.06) 0.22	1.40 (0.79, 2.49) 0.25
Tertile3	2.21 (1.46, 3.33) <0.01	1.98 (1.29, 3.03) <0.01	2.21 (1.24, 3.94) <0.01

In sensitivity analysis, Waist Circumference was converted from a continuous variable to a categorical variable (tertiles).

OR, odds ratio; 95% Cl, 95% confidence interval.

^1^Model 1: No covariates were adjusted.

^2^Model 2: Adjusted for age and race.

^3^Model 3: Adjusted for age, race, education level, Ratio of family income to poverty, marital status, Minutes sedentary activity, Meals from fast food or pizza place, Total Cholesterol, Direct HDL-Cholesterol, hypertension, and diabetes.

Transforming WC from a continuous variable to one stratified by Normal, Overweight, and Obese, comparing waist circumference outside the normal range with that of the normal range revealed that the positive association between WC and infertility prevalence remained stable in the fully adjusted model (model 3) (OR1.02; 95% CI 1.00-1.03). Women with Obese WC were significantly 127% more likely to develop infertility than women with standard WC (**Table S1**).

### Smoothing curve fitting

**Figure 2:**
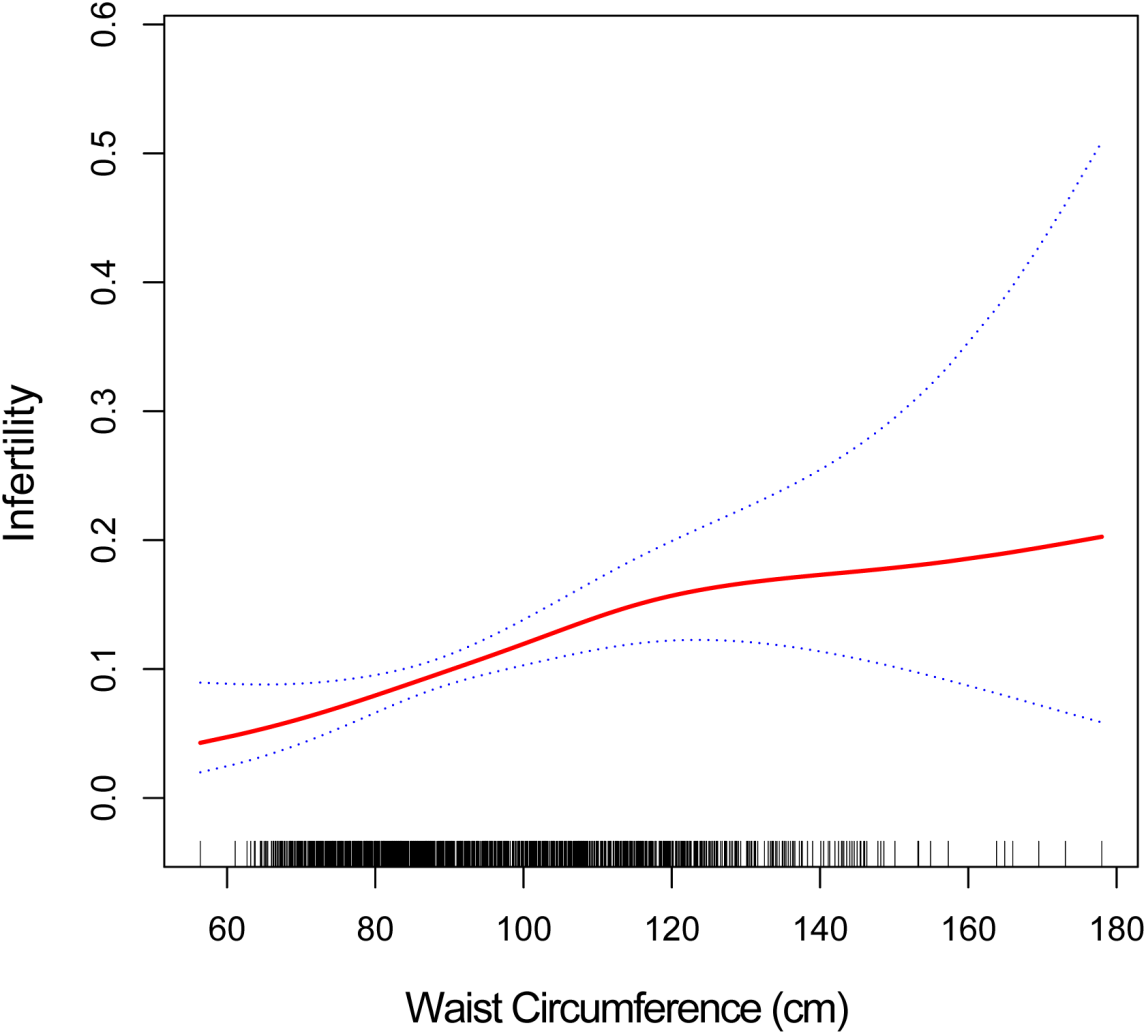
WC and infertility have a positive non-linear connection.

Smoothed curve fitting was used to investigate the nonlinear association between WC and infertility. Our findings show that WC and infertility have a positive non-linear connection (**Figure 2**). The solid red line shows a smooth curve fit between the variables. The 95% fitted confidence interval adjusts for age, household income to poverty ratio, minutes of sedentary activity, eating from fast food or pizza restaurants, and diabetes. Hypertension, high cholesterol levels, total cholesterol, and direct HDL-cholesterol, with results indicated by blue bands.

### Analysis of the threshold effect of WC on the prevalence of infertility

We found a non-linear relationship (log-likelihood ratio *p* < 0.05) by examining the threshold effect of WC on infertility prevalence, and we found that the inflection point for infertility prevalence was 116.6 cm. The adjusted OR for infertility prevalence increased by 3% for each 1-unit increase in waist circumference level when the waist circumference range was less than 116.6 cm (OR1.03; 95% CI 1.01,1.04). When the waist circumference range was greater than 116.6 cm, there was no association with infertility prevalence (OR0.99; 95% CI 0.97,1.02) (**Table 3**). For this phenomenon, we hypothesize that it may be an effect of extreme waist circumference values or partial missing samples, and in the future, we will conduct a prospective cohort study using laboratory tests to validate the results in the present study and look forward to more evidence from related studies to confirm it.

**Table 3:** Analysis of the threshold effect of WC on the prevalence of infertility.

	Adjusted OR (95%CI), *p* value
Infertility	
Fitting by the standard linear model	1.02 (1.01, 1.03) 0.0025
Fitting by the two-piecewise linear model	
Inflection point	116.6cm
Waist Circumference <	1.03 (1.01, 1.04) <0.01
Waist Circumference >	0.99 (0.97, 1.02) 0.54
*p* for Log-likelihood ratio	0.043

The results of the threshold effect of WC on the prevalence of infertility was adjusted for age, Ratio of family income to poverty, Minutes sedentary activity, Meals from fast food or pizza place, Diabetes, Hypertension, high cholesterol level, Total Cholesterol, Direct HDL-Cholesterol.

### Subgroup analysis

For the association between WC and infertility, the upper panel observed a positive association among participants stratified by Married with a Partner, no diabetes, no hypertension, and no high cholesterol. Among Married with Partner (OR1.02; 95% CI 1.01,1.03) and without hypertension (OR1.02; 95% CI 1.00,1.03), each unit increase in WC was associated with a 2% increase in the likelihood of infertility. Among those without diabetes (OR1.01; 95% CI 1.00,1.02) and without high cholesterol (OR1.01; 95% CI 1.00,1.03), each unit increase in WC was associated with a 1% increase in the likelihood of infertility (**Table 4**).

**Table 4:** Subgroup analysis.

Infertility	OR (95%CI)	*p* for interaction
Marital status(%)		0.3757
Married with Partner	1.02 (1.01,1.03)	
Divorced	0.99 (0.95,1.05)	
Never married	1.00 (0.98,1.03)	
Diabetes(%)		0.358
Yes	1.03 (0.99,1.07)	
No	1.01 (1.00,1.02)	
Hypertension(%)		0.521
Yes	1.00 (0.96,1.04)	
No	1.02 (1.00,1.03)	
high cholesterol level(%)		0.9199
Yes	1.01 (0.98,1.04)	
No	1.01 (1.00,1.03)	

OR, odds ratio; 95% CI, 95% confidence interval.

The interaction term did not report the effect of marital status, diabetes, hypertension, and high cholesterol on the association between WC and infertility (*p* for all interaction tests > 0.05).

## Discussion

In this cross-sectional investigation, which recruited 1,509 female subjects, we discovered a non-linear correlation. A positive non-linear association between waist circumference and increased risk of infertility, with a different relationship between the effect of WC on infertility, reflected the left and right of the inflection point (WC = 116.6). The likelihood of infertility was positively correlated. In contrast, the association on the right side of the inflection point was not statistically significant. Marital status, diabetes, hypertension, and high cholesterol did not have a considerable dependence on this association, suggesting that increased waist circumference could raise the risk of infertility. The findings of this investigation indicate that controlling waist circumference levels can help reduce the risk of infertility.

This is the first study to assess the correlation between WC and female infertility directly. Previous studies have shown that obesity negatively affects reproductive health, mainly regarding reduced fertility and infertility; obese women are more prone than normal-weight women to experience spontaneous abortions and congenital defects in the early stages of pregnancy (25, 26). Obesity has been linked to ovulation issues, and women with a BMI above 27 are more likely to experience anovulatory infertility, which is related chiefly to endocrine reasons (27, 28). Increased peripheral aromatization of estrogen by androgens is a consequence of oxidative stress and ovarian inflammation brought on by obesity. Insulin resistance and hyperinsulinemia also cause hyperandrogenemia, reducing gonadotropin output and responsiveness. In obese women, leptin levels increase while GH and insulin-like growth factor binding protein levels decrease, interfering with the neural regulation of the HPO axis and ovarian function, reducing preimplantation embryo development and uterine tolerance, and increasing the risk of infertility and miscarriage (29, 30). In addition, excessive body obesity worsens polycystic ovary syndrome and further becomes an essential fundamental of infertility for ovulation disorder. According to research, ovulatory variables account for 25% of infertility cases. Polycystic ovarian syndrome (PCOS), the most prevalent cause of anovulation that affects about 70% of anovulatory women, may be improved by significant weight loss to lowering insulin sensitivity (31).

In a recent clinical trial, obese and infertile women received a six-month lifestyle modification from Vincent Wekker et al. Women in the intervention group had improved sexual function and more vaginal lubrication than women in the control group, according to the findings of a five-year RCT. That weight loss was also beneficial for cardiovascular health, improved body mass, and infertility (32); Another comparable study revealed that peri-pregnancy weight reduction in obese infertile women might boost conception rates and lower the risk of hypertensive pregnancy problems and premature birth (33). According to José Bellver et al.’s cohort research, obesity impairs the endometrial environment and tolerability by delaying WOI, which causes metabolic abnormalities in women and results in infertility and poor ART outcomes (34).

Waist circumference (WC) is a measurement that is highly correlated with adiposity and has been associated with a variety of adverse pregnancy outcomes, including assisted reproduction failure, gestational diabetes mellitus, gestational hypertension, and preeclampsia; According to a prospective study by Li et al, waist circumference was found to be inversely related to the likelihood of live birth in women undergoing assisted reproductive technology, independent of body mass index (35). According to Taha Takmaz et al., pregnant women who gained weight and had increased WC measures may be at risk for gestational diabetes (36). According to a birth cohort study by Xiao Gao et al., prenatal weight increase and waist circumference are highly linked to unfavorable pregnancy outcomes such as gestational diabetes, primary cesarean delivery, and composite outcomes (one or more adverse pregnancy outcomes) (37). This conclusion is backed up by research by Ariel Zilberlicht et al., who indicated that a healthy pre-pregnancy weight and waist circumference might lower the incidence of unfavorable pregnancy outcomes (38). Wendland EM et al. find that waist circumference predicts adverse pregnancy outcomes associated with obesity (39). In addition, several studies have shown that rising rates of overweight and obesity in obstetric populations put women in greater danger of developing pre-eclampsia, gestational hypertension, and gestational diabetes (40–42). There are more studies on waist circumference and male reproduction but fewer on the correlation between female infertility and waist circumference.

Our research concentrated on the link between female infertility and waist circumference. We observed a positive association between increased waist circumference and increased prevalence of infertility in both the crude and adjusted models. A sensitivity analysis using WC as a tertile also demonstrated a positive association between WC and infertility prevalence. In addition, we found a nonlinear relationship between WC and infertility prevalence by threshold effect analysis with an inflection point of 116.6, with a positive correlation to the left and no correlation to the right of the inflection point. In conclusion, WC has been widely reported as an indicator of obesity events and reproductive disorders, also recognized in our study. According to the study’s findings, It is important for women of reproductive age to be aware of their WC and can find out about this data by consulting their healthcare provider.

There are several advantages of our study. First, our study is based on the NHANES database, and all analyses were considered to select appropriate NHANES sampling weights so the results are more representative. Second, we found for the first time a positive association between WC and the prevalence of infertility in a cross-sectional study of women in the U.S. Through sensitivity analyses, we explored the nonlinear relationship between infertility and WC through smoothed curve-fitting and threshold effect analyses. Third, WC is an easy clinical measure to perform and should be considered to be more routinely incorporated in medical reviews of women of childbearing age to counsel them on their increased risk of infertility. Fourth, our study added two new variables that were strongly associated with obesity and waist circumference, namely Minutes sedentary activity and Meals from fast food or pizza place. Between WC tertile, the individuals with the highest WC tertile compared to other categories were likely to be sedentary women who ate fast food or pizzeria meals frequently.

However, there are some limitations to our study. The first is reflected in age and region; the study sample was 1509 women aged 18-45 years in the United States, so the findings are not informative for male or female patients outside the United States and not in that age range. Second, due to our study’s cross-sectional nature, we could not establish a clear causal link between WC and infertility. Third, the information limitations of the NHANES database prohibit a more thorough analysis of other indicators of infertility, such as immune antibodies, reproductive hormones, and ultrasound, as well as further determining the infertility history and duration of the patients. In addition, the lack of information regarding the diagnosis of polycystic ovary syndrome (PCOS) is a weakness, as this may be the greatest predictor of infertility.

## Conclusion

Our research found that WC was associated with an increased prevalence of infertility. Therefore, high waist circumference values may be a potential risk for infertility. This study will provide support for women and their healthcare providers. Nevertheless, further extensive prospective studies are required to support the results of this article.

## Data availability statement

The datasets presented in this study can be found in online repositories. The names of the repository/repositories and accession number(s) can be found below: https://www.cdc.gov/nchs/nhanes/.

## Ethics statement

The studies involving human participants were reviewed and approved by the NCHS Ethic Review Board. The studies were conducted in accordance with the local legislation and institutional requirements. The participants provided their written informed consent to participate in this study. Written informed consent was obtained from the individual(s) for the publication of any potentially identifiable images or data included in this article.

## Author contributions

Y-HY and A-HW designed the study. Y-HY, S-YZ, D-FL, and X-PC collected, analyzed the data and drafted the manuscript. X-DH, BL, and SL gave suggestions, and Y-HY and A-HW revised the manuscript. All authors contributed to the article and approved the submitted version.

## Supplementary material

Supplementary Table 1 Association of the categorical variable WC with infertility. Classification of WC into groups: Normal(< 80cm); Overweight (80 ~ 88cm); Obese (≥ 88cm). OR, odds ratio; 95% Cl, 95% confidence interval. ^1^Model 1: No covariates were adjusted. ^2^Model 2: Adjusted for age and race. ^3^Model 3: Adjusted for age, race, education level, Ratio of family income to poverty, marital status, Minutes sedentary activity, Meals from fast food or pizza place, Total Cholesterol, Direct HDL-Cholesterol, hypertension, and diabetes.
