# Supplementary material for: Higher waist circumference is associated with increased likelihood of female infertility: NHANES 2017-2020 results
Source: Front Endocrinol (Lausanne). 2023 Oct 20;14:1216413. doi: 10.3389/fendo.2023.1216413 (PMC10627239; doi:10.3389/fendo.2023.1216413)
Supplement: Supplementary Table 1 — Association of the categorical variable WC with infertility. Classification of WC into groups: Normal(< 80cm); Overweight (80 ~ 88cm); Obese (≥ 88cm). OR, odds ratio; 95% Cl, 95% confidence interval. 1Model 1: No covariates were adjusted. 2Model 2: Adjusted for age and race. 3Model 3: Adjusted for age, race, education level, Ratio of family income to poverty, marital status, Minutes sedentary activity, Meals from fast food or pizza place, Total Cholesterol, Direct HDL-Cholesterol, hypertension, and diabetes. [file Table_1.pdf]

TABLE S1 | Association of the categorical variable WC with infertility

	OR(95%CI), *p* value		
	**Model 1[1]**	**Model 2[2]**	**Model 3[3]**
Infertility			
Waist Circumference	1.02 (1.01, 1.02) <0.01	1.02 (1.01, 1.02) <0.01	1.02 (1.00, 1.03) <0.01
Categories			
Normal	Reference	Reference	Reference
Overweight	2.01 (1.11, 3.63) 0.0204	1.80 (0.99, 3.27) 0.0550	2.00 (0.94, 4.25) 0.0702
Obese	2.38 (1.47,3.86) <0.01	2.06 (1.25, 3.39) <0.01	2.27(1.16, 4.45) 0.0165

Classification of WC into groups:Normal（< 80cm）; Overweight (80 ~ 88cm) ; Obese (≥ 88cm)

OR, odds ratio; 95% CI, 95% confidence interval.

[1]Model 1: No covariates were adjusted.

[2]Model 2: Adjusted for age and race.

[3]Model 3: Adjusted for age, race, education level, Ratio of family income to poverty, marital status, Minutes sedentary activity, Meals from fast food or pizza place, Total Cholesterol, Direct HDL-Cholesterol, hypertension, and diabetes.
